# Efficacy of artemether–lumefantrine therapy for the treatment of uncomplicated *Plasmodium falciparum* malaria in Southwestern Ethiopia

**DOI:** 10.1186/s12936-015-0826-9

**Published:** 2015-08-15

**Authors:** Seleshi Kebede Mekonnen, Girmay Medhin, Nega Berhe, Ronald M Clouse, Abraham Aseffa

**Affiliations:** College of Health Siences, Jimma University, Jimma, Ethiopia; Aklilu Lemma Institute of Pathobiology, Addis Ababa University, Addis Ababa, Ethiopia; Armauer Hansen Research Institute, Addis Ababa, Ethiopia; Department of Bioinformatics and Genomics, University of North Carolina at Charlotte, Charlotte, NC USA; Ethiopia and Centre for Imported and Tropical Diseases, Oslo University Hospital-Ulleval, Oslo, Norway

**Keywords:** Artemesinin combination therapy, Treatment failure

## Abstract

**Background:**

The development and spread of chloroquine-resistant *Plasmodium falciparum* threatens the health of millions of people and poses a major challenge to the control of malaria. Monitoring drug efficacy in 2-year intervals is an important tool for establishing rational anti-malarial drug policies. This study addresses the therapeutic efficacy of artemether-lumefantrine (AL) for the treatment of *Plasmodium falciparum* in southwestern Ethiopia.

**Methods:**

A 28-day in vivo therapeutic efficacy study was conducted from September to December, 2011, in southwestern Ethiopia. Participants were selected for the study if they were older than 6 months, weighed more than 5 kg, symptomatic, and had microscopically confirmed, uncomplicated *P. falciparum*. All 93 eligible patients were treated with AL and followed for 28 days. For each patient, recurrence of parasitaemia, the clinical condition, and the presence of gametoytes were assessed on each visit during the follow-up period. PCR was conducted to differentiate re-infection from recrudescence.

**Results:**

Seventy-four (83.1 %) of the study subjects cleared fever by day 1, but five (5.6 %) had fever at day 2. All study subjects cleared fever by day 3. Seventy-nine (88.8 %) of the study subjects cleared the parasite by day 1, seven (7.9 %) were blood-smear positive by day 1, and three (3.4 %) were positive by day 2. In five patients (5.6 %), parasitaemia reappeared during the 28-day follow-up period. From these five, one (1.1 %) was a late clinical failure, and four (4.5 %) were a late parasitological failure. On the day of recurrent parasitaemia, the level of chloroquine/desethylchloroquine (CQ-DCQ) was above the minimum effective concentration (>100 ng/ml) in one patient. There were 84 (94.4 %) adequate clinical and parasitological responses. The 28-day, PCR-uncorrected (unadjusted by genotyping) cure rate was 84 (94.4 %), whereas the 28-day, PCR-corrected cure rate was 87 (97.8 %). Of the three re-infections, two (2.2 %) were due to *P. falciparum* and one (1.1 %) was due to *P. vivax*. From 89 study subjects, 12 (13.5 %) carried *P. falciparum* gametocytes at day 0, whereas the 28-day gametocyte carriage rate was 2 (2.2 %).

**Conclusions:**

Years after the introduction of AL in Ethiopia, the finding of this study is that AL has been highly effective in the treatment of uncomplicated *P. falciparum* malaria and reducing gametocyte carriage in southwestern Ethiopia.

## Background

Although there have been encouraging reports of declining of morbidity and mortality from malaria in most endemic countries [1], it remains an overwhelming public health problem. At present, approximately 198 million people have malaria worldwide, leading to 5,84,000 deaths per year. Ninety percent of these deaths are in Africa, and approximately 453,000 of those are children under five children [2].

In Ethiopia 75 % of the area is malarious, and approximately 52 million people live in high-risk areas, mainly at altitudes below 2,000 metres [3]. *Plasmodium falciparum* and *P. vivax* are the two dominant parasite species, with relative frequencies of about 60 and 40 %, respectively (although thosevary by location and season). Still, *Plasmodium falciparum* is the dominant parasite species, causing severe and complicated manifestations and is responsible for most malarial deaths [4].

Early diagnosis and effective treatment are essential elements for the control of malaria. However, one of the obstacles to controlling malaria is the capability of the parasites to evolve resistance to various anti-malarial drugs*. Plasmodium falciparum* has an extraordinary ability to do this, creating a major challenge for the control efforts and increasing the number of deaths in sub-Saharan Africa [5, 6].

In Ethiopia, resistance of *P. falciparum* to a standard triple-dose of chloroquine (25 mg base/kg) [7], and a report from Debre Zeit of chloroquine (CQ) treatment failure for *P. falciparum* and *P. vivax*, brought a policy change, and sulfadoxine-pyrimetamine (SP) has been used as an affordable alternative treatment of uncomplicated malaria cases since 1998 [8]. Unfortunately, unlike chloroquine, SP was used extensively, and parasites developed resistance within a short time [9]. Moreover, earlier studies reported widespread and high rates of therapeutic failure to SP for the treatment of *P. falciparum* [10]. As a result, in 2001, WHO recommended a shift to artemisinin-based combination therapy (ACT) for the treatment of *P. falciparum* malaria for all countries experiencing resistance to monotherapies in 2001 [11]. Accordingly, Ethiopia replaced SP by artemether–lumefantrine (AL), which showed no treatment failure and no significant after a follow-up period of 14 days in 2004 [12]. Replacing ineffective anti-malarial drugs with ACT has reduced the morbidity and mortality associated with malaria. SP is still used, however, as intermittent preventive therapy for pregnant women in Ethiopia [13].

ACT was designed to attack malaria parasites with different mechanisms of action simultaneously. It reduces the emergence of drug resistance, and it gives a faster relief from clinical symptoms and parasite clearance [14]. Artemether is quickly hydrolyzed to dihydroartemisinin (DHA), its main active metabolite, and absorbed rapidly. Artemether and DHA reduce asexual parasite mass by 10,000-fold per reproductive cycle. The partner drug, lumefantrine, is absorbed and cleared more slowly, acting to eliminate the remaining parasites and thus prevent recrudescence [15]. Twice-daily dosing of AL for 3 days maintains artemether and DHA concentrations at supratherapeutic levels [16].

To insure timely changes to treatment policy, WHO recommended therapeutic efficacy studies of the drug every 2 years [5]. Accordingly, a study of AL was conducted in Kersa, and it reported excellent therapeutic efficacy [17]. However, in 2006 reports of higher recrudescence rates of *P. falciparum* malaria after treatment with ACTs emerged from observational data collected in Cambodia [18]. Moreover, another study carried out in 2006 and 2007 in Battambang province, Cambodia, showed delayed parasite clearance times [19]. Delay in parasite clearance and subsequent treatment failure is multifactorial in origin, with the host, parasite, and drug factors contributing almost equally. As a consequence, there has been an increasing concern about the delay in parasite clearance with artemesinin in Southeast Asia Since 2009 [20].

The development of resistance to ACT could have a major impact on malaria control efforts at time when there are no other drugs in the development pipeline. Therefore, the primary aim of this study was to assess the therapeutic efficacy of AL for the treatment of uncomplicated *P. falciparum* malaria in Southwestern Ethiopia. The secondary objectives of the study were to determine the prevalence of post-treatment gametocyte carriage in Southwestern Ethiopia. This information will inform policy makers with respect to appropriate antimalarial strategies.

## Methods

**Fig. 1:**
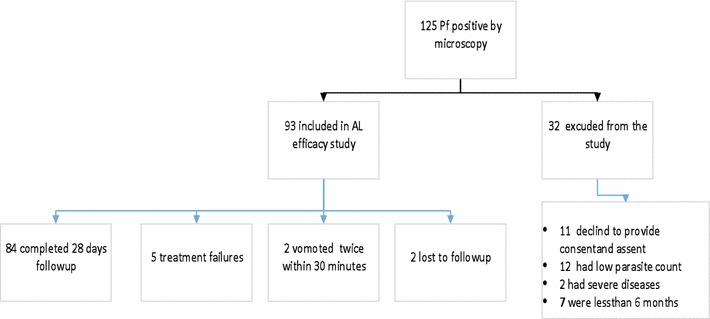
Study subjects participated on AL efficacy study and treatment outcome. From a total of 125 *P. falciparum* cases, 93 fulfilled the inclusion criteria and gave written and informed consent and assent to participate in the therapeutic efficacy of AL for the treatment of *P. falciparum,* while 32 study subjects did not meet the inclusion criteria and were excluded from the study. From 32 excluded from the study, 11 excluded to provide consent and assent, 12 had a low parasite count from the thick blood film, 2 had sever disease (Tb positive) and 7 were less than 6 months. Among the 93 study participants, one blood film reading was discrepant between the two microscopic readers and decision was made based on the third senior laboratory personnel reading. On the first day of the study, two study subjects vomited the drug twice and excluded from the study and analysis. On day 14 and 21, two patients did not return on the follow-up day. We could not find one of the patients, and we were informed that the other patient had moved from the area, so both were considered lost to follow-up.

This study was conducted between August and December (the malaria season), 2001, at Omo Nada health center in southwestern Ethiopia. Following WHO protocols [21], we evaluated the clinical and parasitological responses to the treatment of uncomplicated *P. falciparum* malaria with artemether–lumefantrine (Fig. 1).

### Study area, period and design

Omo Nada (7°37′60″N, 37°15′0″E) is one of the woredas (the name for districts in Ethiopia) in Jimma Zone, Oromia Region of Ethiopia. Nada is the administrative center of the woreda; other towns in Omo Nada include Asendabo. The altitude of this woreda ranges from 1,000 to 3,340 meters above sea level, and its total population is 2,48,173. As in most other places, malaria transmission in Omo Nada follows rainy seasons, with transmission peaking in the months between September and December and between April and May. The main malaria control strategy in the woreda includes use of Long Lasting Insecticidal Nets (LLINs), malaria case management with ACTs, and intermittent preventive treatment during pregnancy (IPTp) [22].

### Sample size determination

For therapeuthic efficacy studies, sample sizes (n) were calculcated based on recommendations by the Technical Expert Group on Malaria Chemotherapy [21], using the formula n = (Z/d)^2^ P (1 − P), where P stands for the anticipated prevalence, d for the margin of error, and Z for the Z statistic at a given level of confidence. For the level of confidence of 95 %, the Z value is 1.96. In this study a 5 % failure rate was expected, the precision level was set a 4.5 %, and a loss-to-follow-up rate was anticipated to be 20 % over 28-days. Thus the calculated sample size was 93, which was considered sufficient to address the study objectives.

### Study subjects and inclusion criteria

The study subjects were recruited among *P. falciparum*-positive patients visiting health centres who met the WHO inclusion guidelines for the assessment and monitoring of anti-malarial drug efficacy for the treatment of uncomplicated malaria [5]. The details of inclusion and exclusion criteria are presented as follows:

The inclusion criteria were as follows:

- Over 6 months of age
- Living in areas of low-to-moderate transmission
- Mono-infection with *P. falciparum* detected by microscopy
- Asexual parasite count of 1,000–100,000/µl in areas of low-to-moderate transmission
- Axillary temperature ≥37.5 °C or history of fever during the 24 h before recruitment
- Ability to swallow oral medication
- Ability and willingness to comply with the protocol for the duration of the study and to comply with the study visit schedule
- Informed consent from the patient (or parent or guardian in the case of children, and assent from children between 7 and 17 years)

The exclusion criteria were as follows:

- General danger signs in children under 5 years (coughing or difficulty breathing) or signs of severe *P. falciparum* malaria, according to the definitions of WHO
- Severe malnutrition according to WHO growth standards (defined as follows: for children 6 months to 18 years, a growth standard z-score below −3, symmetrical oedema involving at least the feet or a mid-upper arm circumference of <110 mm; for adults, a mid-upper arm circumference of <170 mm, BMI <16 with or a mid-upper arm circumference <180 mm with recent weight loss or underlying chronic illness).
- Febrile condition due to diseases other than malaria (e.g., measles, acute lower respiratory tract infection, severe diarrhea with dehydration) or other known underlying chronic or severe diseases (e.g., cardiac, renal or hepatic diseases, HIV/AIDS)
- Regular medication which might interfere with anti-malarial pharmacokinetics (like antiretroviral drugs)
- History of hypersensitivity reactions to any medicine tested or used as an alternative treatment or contraindication to AL
- Pregnant or breastfeeding women

### Blood film management during each follow up days

Thick and thin blood smears were prepared and stained with 10 % Giemsa for 10 min. The stained blood films were examined on days 0, 1, 2, 3, 7, 14, 21, and 28, as well as on any unexpected visits with the patient due to the patient suffering a malaria attack before the day of the next appointment. Two laboratory personnel read the blood film slide independently. Blood smears with discordant results (differences between the two microscopists in species diagnosis, or differences in parasite density of >50 %) were re-examined by a third, independent microscopist, and parasite density was calculated by averaging the two closest counts. A blood film was considered negative when no parasite was found after examining 100 fields. On day 0, hemoglobin was determined for those study subjects who were willing to participate on the study. Parasite count was based on the number of asexual parasites observed against 200 leukocytes. This number was then multiplied by 40 to gain an approximate count per microlitre.

$${\text{No}}{\text{. parasites per}}/\upmu L = {\text{Parasite count}}\, \times \,8{,}000/200\,{\text{WBC}}$$

Parasite densities for all participants were calculated using an assumed WBC of 8.0 × 10(9)/L of blood, which has been set by WHO to be used for convenience in facilities which lack the tools to determine patients’ absolute full blood cell count (FBC) values. In addition, the whole Giemsa-stained thick film was examined for gametocyte carriage before and after artemether–lumefantrine treatment on all follow up days.

### Treatment of *Plasmodium falciparum* with AL and follow-up

As a routine procedure within the health system of Ethiopia, study subjects were treated with AL (Artefan) (Ajanta pharma limited, Charkop, Kandivli (W), Mumbai 400 067, India) twice daily on days 0, 1, and 2. Study medication was administered based on weight; the first dose were administered under the supervision of a qualified member of the study staff. Study subjects were followed for 30 min post treatment as an additional procedure from the routine ones. If vomiting occurred, a second full dose was administered. If repeated vomiting occurred, patients were withdrawn from the study and offered rescue therapy. Patients who failed to respond to AL treatment during the 28 days of follow-up were treated with oral quinine (8 mg/kg per day for 7 days), which is a second line of drugs and a regular treatment regime for the treatment of malaria in Ethiopia.

Patients were asked to return to the health centre on days 1, 2, 3, 7, 14, 21, 28 or if suffering from a malaria attack before the day of the next appointment. On each follow-up day, blood was examined for the presence or absence of parasites, and temperature was measured. Those patients with recurrence of parasites during the follow-up days were treated with quinine. Patients were considered lost to follow-up when they did not come to the clinic as scheduled and became unreachable.

### DNA extraction and molecular detection

DNA was isolated using QIAgen DNA Mini Kit for blood and tissue (QIAGEN, Germany), based on the manufacturer’s instructions, and stored at −20 °C until use. A nested PCR assay was then carried out as previously described elsewhere [23].

The most widely used genetic markers for malaria genotyping—the antigen genes *msp1*, *msp2* and *glurp*—were used in this here; these markers have been shown to have adequate discriminatory power for recrudescence *versus* re-infection testing [24]. Merozoite surface protein 1 (msp1) alleles of *P. falciparum* can be divided into 17 distinct blocks. Exept block 2 region, the non-conserved sequences can be grouped into major families represented by the MAD20 [25]. Allelic polymorphism in block 2 was widespread with over 50 different variants. However, these sequences fall into three main types represented by K1, MAD20, and RO33 isolates [26]. The msp2 alleles are grouped into two allelic families, FC27 and 3D7/IC1. Msp2 is highly polymorphic because of intragenic repeats [5, 27]. All the primer sequences were adopted from the Worldwide Antimalarial Resistance Network WWARN (2005) (Table 1) [5].

**Table 1:** Specific primers for *msp1* and *msp2* primer sets

Locus	Allele	Primer		Sequence (5′ to 3′)
		Forward	Reverse	
*msp1*	N/A	✔		CTAGAAGCTTTAGAAGATGCAGTATTG
			✔	CTTAAATAGTATTCTAATTCAAGTGGATCA
	K1	✔		AAATGAAGAAGAAATTACTACAAAAGGTGC
			✔	GCTTGCATCAGCTGGAGGGCTTGCACCAGA
	MAD20	✔		AAATGAAGGAACAAGTGGAACAGCTGTTAC
			✔	ATCTGAAGGATTTGTACGTCTTGAATTACC
	RO33	✔		TAAAGGATGGAGCAAATACTCAAGTTGTTG
			✔	CATCTGAAGGATTTGCAGCACCTGGAGATC
*msp2*	N/A	✔		ATGAAGGTAATTAAAACATTGTCTATTATA
			✔	ATATGGCAAAAGATAAAACAAGTGTTGCTG
	FC27	✔		GCAAATGAAGGTTCTAATACTAATAG
			✔	GCTTTGGGTCCTTCTTCAGTTGATTC
	3D7/IC	✔		AGAAGTATGGCAGAAAGTAAKCCTYCTACT
			✔	GATTGTAATTCGGGGGATTCAGTTTGTTCG

All the primer sequences were adopted from Worldwide Antimalarial Resistance Network, WWARN (2005).

### Study endpoints

Treatment outcomes for AL were determined based on the WHO classification of treatment outcomes as follows: (1) early treatment failure (ETF), (2) late clinical failure (LCF), (3) late parasitological failure (LPF), and (4) adequate clinical and parasitological response (ACPR) [21].

### Ethical clearance

Ethical clearances were obtained from Aklilu Lemma Institute of Pathobiology (ALIPB), Armauer Hansen Research Institute (AHRI/ALERT) and Jimma University before commencing the projects. The Investigator explained the study to each potential subject verbally, provided all the information (purpose, procedures, risks, benefits, alternatives to participation, etc.), and gave time for any queries. Then the potential subject was provided with a written consent form and afforded sufficient time to read it. Once an individual had all his/her questions answered and agreed to participate in the study, they or their family or guardian signed an informed consent prior to inclusion in the study. Confidentiality of information and freedom to withdraw from the study anytime were guaranteed. In addition, study subjects were compensated for transportation costs during the 28-day follow-up period.

## Results

### Study subject participated on AL efficacy study

Among 150 *P. falciparum*-positive patients visiting Omo Nada health centers, 93 fulfilled the inclusion criteria and were included on the AL efficacy study. Of these, two vomited twice within 30 min, and two were lost to follow-up; thus these were excluded from the study.

### Patient characteristics

The majority of the study subjects (59.8 %) were male. The mean age and temperature of the study subjects were 17.3 years and 37.7 °C, respectively. The minimum haemoglobin level was 8.0 g/ml, while the maximum haemoglobin level was 14.6 g/ml. The mean parasite load counted from the thick blood film was 8,404 (2,960–18,400) (Table 2).

**Table 2:** Baseline characteristics of study subjects involved in AL the efficacy study

Patient characteristics	Value
Gender (M %/F %)	59.8 %/40.2 %
Mean age (Year)	17.3 (1–60)
Mean temperature, axillary (°C)	38.8 °C (37.5–40.0 °C)
Mean hemoglobin (gm/ml)	11.6 g/ml (8.0–40.0 gm/ml)
Parasite load (µl)	8,404 (2,960–18,400)
Weight (kg)	34.4 kg (6–69 kg)

### Fever and parasite clearance

Seventy-four (83.1 %) of the study subjects cleared fever by day 1, ten (11.2 %) still had fever on day 1, and five (5.6 %) had fever on day 2. All study subjects cleared fever by day 3. In addition, 79 (88.8 %) of the study subjects cleared the parasite by day 1, seven (7.9 %) were blood-smear positive by day 1, and three (3.4 %) were positive by day 2 (Table 3).

**Table 3:** Patient recruitment and follow up for 28 days

Characteristics	n
Fever clearance	
Fever present on day 1	10 (11.2)
Fever present on day 2	5 (5.6)
Fever present on day 3	0 (0.0)
Parasite clearance	
Smear positive on day 1	7 (7.9)
Smear positive on day 2	3 (3.4)
Smear positive on day 3	0 (0.0)
Appearance of gametocytes	
Gametocyte carriage on day 0	12 (13.5)
Gametocyte carriage on day 28	2 (2.2)

### Treatment outcome during the 28-day follow-up period

Among 89 patients who were included in this study, five (5.6 %) were classified as treatment failures. One (1.1 %) was a late clinical failure, and four (4.5 %) were late parasitological failures. From the four late parasitological failures, parasites were identified from three study subjects on day 14 and from one study subjects on day 21. There were 84 (94.4 %) adequate clinical and parasitological responses. PCR uncorrected cure rate (unadjusted by genotyping) was 94.4 % (95 % CI 88.0–97.9 %) and the PCR corrected cure rate was 97.8 % (95 % CI 92.8–99.6 %). Of the three re-infections, two (2.2 %) were due to *P. falciparum* and one (1.1 %) was due to *P. vivax* (Table 4).

**Table 4:** Treatment outcome during 28 days follow up period

Treatment outcome	N (%)
Treatment failure	
Late clinical failure	1 (1.1)
Late parasitological failure	4 (4.5)
Adequate clinical and parasitological response	84 (94.4)
28 days PCR uncorrected cure rate	84 (94.4)
28 days PCR corrected cure rate	87 (97.8)
Re-infection	
Recurrent malaria by *P. vivax*	1 (1.1)
Recurrent malaria by *P. falciparum*	2 (2.2)
Recrudescence	2 (2.2)

There were 33 study subjects ≤5 years of age and the rest (56) were above 5 years of age. In ≤5 years of age the PCR-uncorrected cure rate was 97.0 % (95 % CI 86.0–100.0 %) while PCR-corrected cure rate was 100.0 % (95 % CI 91.3–0.0 %). On the other hand, PCR-uncorrected and corrected cure rates in study subjects above 5 years of age were 92.9 % (95 % CI 83.7–97.7 %) and 96.4 % (95 % CI 88.7–99.4 %) respectively (Table 5).

**Table 5:** Treatment outcome during 28 days follow up period in different age group

Outcome	≤5 years (N = 33)	>5 years (N = 56)	Total (N = 89)
LCF	0	1 (1.8)	1 (1.1)
LPF	1 (3.0)	3 (5.4)	4 (4.5)
ACPR	32 (97.0)	52 (92.9)	84 (94.4)
28 days PCR uncorrected cure rate	32 (97.0)	52 (92.9)	84 (94.4)
28 days PCR corrected cure rate	33 (100.0)	54 (96.4))	87 (97.8)

### Gametocyte determination using microscopy

The whole thick blood films were examined for the presence of gametocyte stages. From 89 study subjects, 12 (13.5 %) carried *P. falciparum* gametocytes at day 0, whereas the 28 days gametocyte carriage rate was 2 (2.2 %) (Table 3).

## Discussion

The remarkable ability of *P. falciparum* to evolve resistance to a number of anti-malarial drugs has recently increased the number of deaths from malaria worldwide [6, 28]. Chloroquine-resistant *P. falciparum* [7] and high rates of failure to SP [10, 29] have been some of the challenges to combatting malaria in Ethiopia. In response, Artemether–lumefantrine (AL), one of the artemesinin combination therapy (ACT) regimens recommended as first line drug by the World Health Organization in 2001, has been used and proved efficacious for the treatment of uncomplicated *P. falciparum* malaria since 2004 in southwestern Ethiopia. All 93 *P. falciparum* mono-infections in our study received AL, and 74 of them (83.1 %) resolved their fever by day 3. AL is known to clear fevers in a very short period of time (less than 3 days). The parasite clearance in this study was faster than the result reported from Thai–Cambodian border where the parasite resistance was characterized in this efficacy study [30].

Unfortunately, the description of artemisinin resistance in Southeast Asia [20] and the China–Myanmar border [31] is a global concern. Failure to rapidly clear parasites will compromise the use of artemisinin for the treatment of severe malaria. Slow parasite clearance in patients treated with an ACT causes more parasites to be exposed to the partner medicine alone, increasing the risk of resistance developing to the partner medicine. If this occurs, treatment failures are likely to increase [32]. Among the five treatment failures, one (1.1 %) was a late clinical failure and four (4.5 %) were late parasitological failures. From the four late parasitological failures, parasites were identified from three study subjects on day 14 and from one study subjects on day 21. All the four late parasitological failures were treated with quinine, the standard second-line drug for the treatment of *falciparum* malaria in Ethiopia. In the current study there were 84 (94.4 %) adequate clinical and parasitological responses. That means PCR uncorrected cure rate (unadjusted by genotyping) was 94.4 % (95 % CI 88.0–97.9 %) and The PCR corrected cure rate was 97.8 % (95 % CI 92.8–99.6 %). It is concluded that the efficacy of AL for the treatment of uncomplicated *falciparum* malaria was high in this study.

Likewise, the high efficacy of AL has been reported in earlier studies from Ethiopia [33–35]. Another study from southern Ethiopia reported a 97.2 % cure rate and showed that the drug could continue as a first-line treatment [36]. Moreover, high efficacy of AL in the treatment of uncomplicated malaria was confirmed by Seboxa et al. [5] in their study from southwestern Ethiopia and the finding of this study is in agreement with reports of high efficacy of AL in East African countries [37, 38]. The reason for the efficacy of AL was its rapid killing of the sexual stages of *P. falciparum* and the activity of AL on gametocytes. Modeling studies have suggested that the spread of anti-malarial drug resistance is primarily driven by a “window of selection” after therapy, and the duration of this window is increased for drugs with longer elimination half-lives. The artemisinin drugs have short elimination half-lives and are thus less likely to select for resistance [39, 40]. Moreover, in the Ethiopian context, the distribution of AL only in the government sectors (organizations) has minimized the distribution of counterfeit drugs, which have already penetrated the African market [41]. In Cambodia, the epicentre for anti-malarial drug resistance, the cheaper “artesunate” contains 6 % chloroquine but no artesunate, and the cheaper “mefloquine” contains sulfadoxine-pyrimethamine but no mefloquine. Chloroquine and sulfadoxine-pyrimethamine are ineffective in this area [42].

In the current study the whole thick blood films were examined for the presence of the gametocyte stage, and there was a signifiant reduction of gametocyte carriage at day 28. Gametocyte carriage reduction after treatment was also seen in children with uncomplicated *P. falciparum* malaria in southwestern Nigeria: fter treatment with AL, 8, 1, 1, 3, 3, and 3 children were gametocyte carriers on days 0, 3, 7, 14, 21, and 28, respectively [43]. In addition, a study in Thailand showed that when artemisinin derivatives were introduced as a component of first-line treatment, there was a significant reduction in the incidence of clinical *P. falciparum* malaria during the next 2 years [44]. Furthermore, artemisinin is active against the broadest range of stages in the life cycle of *Plasmodium* species and kills gametocytes, the sexual stage of the malaria parasite responsible for infection of the mosquito. Artemisinins also kill immature and developing gametocytes, the sexual stages that are essential for transmission, thereby reducing transmission and contributing to malaria-control programs [45].

One of the limitations of this study was AL was given without fatty food. As well known, food enhances the oral absorption of artemether and lumefantrine. In healthy volunteers, the relative bioavailability of artemether increased by two- to threefold and that of lumefantrine by 16-fold when administered after a high-fat meal, as opposed to under fasting conditions [46].

## Conclusions

Eight years after the introduction of AL, this efficacy study revealed that it has been highly effective in the treatment of uncomplicated *P. falciparum* malaria and reducing gametocyte carriage in southwest Ethiopia. The result of this study supports the continuation of AL as a first line treatment for uncomplicated malaria, and there is presently no threat of artemisinin resistance developing in Southwest Ethiopia. This result can perhaps be generalized to areas with similar setting in East Africa.
